# Prolonged Hospitalisation After Lung Cancer Surgery: A Qualitative Study of Patient Experiences of the Reasons for Their Extended Stay

**DOI:** 10.1155/nrp/5710363

**Published:** 2026-09-25

**Authors:** Pernille Orloff Donsel, Malene Missel

**Affiliations:** ^1^ Department of Cardiothoracic Surgery, Heart Centre, Rigshospitalet, Copenhagen University Hospital, Copenhagen, Denmark, gentoftehospital.dk; ^2^ Department of Clinical Medicine, Copenhagen University, Copenhagen, Denmark, ku.dk

## Abstract

**Aim:**

To explore patient experiences of the reasons for prolonged hospitalisation after video‐assisted thoracoscopic surgery lobectomy in an enhanced recovery after surgery programme, when no medical reasons have been identified for the extended stay.

**Design:**

Qualitative study.

**Methods:**

Nine unstructured face‐to‐face interviews were conducted with patients who had undergone lung cancer surgery and remained hospitalised despite having recovered somatically.

**Results:**

Guided by Gadamer’s philosophical hermeneutical approach, the analysis identified the following three themes regarding patient experiences: role as patient or caregiver, ready to be discharged and relatives as a shield against worry.

**Conclusion:**

Patients who have undergone lung cancer surgery in an enhanced recovery after surgery programme are often discharged as planned. However, some patients are in such different situations that it is not always possible for them to meet criteria for a standard time for hospital discharge. Possible factors that can lead to a decision to prolong hospitalisation include patient circumstances at home, e.g., caring for a sick partner, the healthcare professionals’ awareness of the patient’s general situation and family members who can give the patient support and reassurance.

**Implications for the Profession and Patient Care:**

Healthcare professionals can use the findings to understand why patients sometimes stay in the hospital even though they seem medically ready for discharge. Well‐established guidelines may inadvertently overshadow the patients’ needs, and healthcare professionals must take an individual approach to patients when discharge planning.

**Reporting Methods:**

Consolidated Criteria for Reporting Qualitative Research guidelines were followed.

## 1. Introduction

Video‐assisted thoracoscopic surgery (VATS), which is less invasive than lobectomy via thoracotomy, is widely recognised for transforming the way lung cancer surgery is practiced, with VATS lobectomy now considered the gold standard for curative treatment of early‐stage lung cancer [1]. Use of the VATS approach has led to changes in the postoperative trajectory and nursing care based on Enhanced Recovery After Surgery (ERAS), which is a multimodal care pathway designed to achieve early recovery for patients undergoing major surgery [2]. ERAS specifically focuses on early mobilisation, early drainage removal, sufficient nutrition and pain treatment [3]. This approach to structuring surgical treatment and postoperative care benefits patients in various ways, resulting in, for example, less pain, less loss of muscle mass, a lower surgical stress response and shorter hospitalisation.

A single‐centre prospective consecutive cohort study that included 147 patients showed that the mean hospital stay at the Department of Thoracic Surgery, Rigshospitalet, Denmark, after VATS lobectomy was two days postsurgery [4]. However, 69 of the patients in the study had a prolonged hospital stay (> 2 days) due to postoperative complications. Important factors for prolonged hospitalisation were air leak (27.7%), pneumonia (20.2%), pain (15.3%), urinary/renal factors (11.0%), atrial fibrillation (7.0%), respiratory failure (4.5%), cognitive factors/delirium (4.3%), gastrointestinal factors (3.8%), oxygen dependency (2.7%), social factors (2.0%) and pleural effusion (1.4%) [4].

In *What’s going on in the hospital?: Investigating patient experiences of a prolonged in-hospital stay after VATS lobectomy*, Donsel and Missel [5] explored how patients experienced and coped with prolonged hospitalisation due to medical complications in an ERAS programme. The patients included in the study did all have a medical reason for staying in hospital. However, Huang et al. [4] found that nonmedical reasons, i.e., social factors, could also lead to delayed discharge, but exactly what those factors cover for the patient remains unknown. As a result, this qualitative study aims to explore the term *social factors*, defined as ‘*any factor without association to health, for example, living alone, or waiting for a home transportation*’ ([4], p. 2), by examining patients’ experiences of why they remain in hospital when medically ready for discharge.

## 2. Background

Lung cancer continues to be the leading cause of cancer‐related deaths worldwide, with the highest mortality rates among both men and women [6]. The most common types of lung cancer are non–small cell carcinoma and small cell carcinoma, with the former being the most common and slower growing than the latter, which is less common and faster growing. An estimated 1.8 million people died of lung cancer in 2020, making it a significant public health concern worldwide [6]. However, Denmark has seen a significant increase in 5‐year survival rates. From 1982 to 1986, the 5‐year survival rate was 5.9% for women and 5.8% for men, and in 2017–2021, it was 32.1% for women and 25.2% for men [7]. One partial explanation for the increased survival rate is procedural packages [8], which, in Denmark, describe the organisational structure, criteria for initiation of packages and initial treatment for patients suspected or diagnosed with cancer. In addition, better treatment options, including a breakthrough in surgical treatment with VATS and ERAS, have had a significant impact on patient outcomes and survival [8, 9].

Patients undergoing lung cancer surgery now spend less time in hospital on average than before due to VATS and ERAS. However, there are still cases where hospitalisation lasts longer than expected. In a Danish context, Huang et al. [4] found that 47% of patients who underwent VATS lobectomy were hospitalised longer than the average of 2 days. Rojas‐García et al. [10] identified delayed discharge as ‘*the period of continued hospital stay after a patient is deemed medically fit to leave hospital but is unable to do so for non-medical reasons*’ (p. 41). Their study found that delayed discharge was associated with greater mortality, infections and depression but also reduced mobility and daily activities. Furthermore, they found that delayed discharge affects the patients’ emotional state due to noise, lack of personal privacy and limited autonomy. Finally, the study found that patients expressed anxiety and negative feelings such as boredom, depression and loss of independence when discharge was delayed. Overall, Rojas‐García et al. found that prolonged hospitalisation, despite the patient being medically ready, can have various negative consequences for patient well‐being.

A 3‐year, single‐centre retrospective study that included 250 patients found that patient‐reported poor preoperative quality of life is a predictor of prolonged in‐hospital stay after VATS lobectomy for lung cancer [11]. Pompili et al.’s study concluded that baseline quality of life status should be considered when implementing psychosocial supportive programmes when patients are enrolled in an ERAS programme. In a different study exploring factors that affect readiness for discharge in patients with total hip replacement in ERAS programmes, Heine et al. [12] examined the patients’ experiences and concluded that their main concern was feeling safe. Patients who felt safe perceived that they were ready to be discharged. Their study also suggested that healthcare professionals can recognise patient concerns prior to discharge to promote good patient care and discharge planning that patients find more acceptable.

A qualitative study examining nursing care practices for patients with lung cancer who remain hospitalised despite having recovered somatically found that prolonging hospitalisation depended on clinical judgement [13]. The study’s analysis of 16 nurses who participated in qualitative focus group interviews showed that when patients are enrolled in ERAS programmes, a counterculture emerged that contrasted with the logic of productivity, indicating that caring as a worthwhile means in itself may be underestimated in protocol‐driven care. The study further concluded that the aim of nurses is not to keep patients in the hospital longer but to avoid needless suffering [13].

The studies mentioned above show that hospitalisation can be prolonged for various reasons and factors at different points during treatment. The last‐mentioned study showed how nurses navigate hospitalisation time to avoid needless suffering, but we did not find any studies examining patient experiences of why they remain hospitalised despite being medically ready for discharge. Therefore, the aim of this qualitative study is to explore patient experiences of the factors contributing to their prolonged hospitalisation, defined as a situation where no medical reason is identified for their extended stay.

## 3. Methods

### 3.1. Study Design

This study, which explores the experiences of patients who are undergoing treatment for lung cancer, uses a methodological approach considered appropriate for obtaining lengthy and detailed descriptions of the patient perspective. The study relies on Gadamer’s [14] philosophical hermeneutics approach to understanding, which asserts that people have historically conditioned preconceptions that determine what can be experienced. This means that people’s preconceptions should not be seen as an impediment but as a prerequisite for their ability to gain a new understanding. As clinical nurse specialists in thoracic surgery and researchers in the field of nursing care for patients with lung cancer and their care and treatment trajectory, we have experience with discharge processes and patients’ transition from hospital to home. These experiences shape the questions we are able to ask. While this knowledge provides valuable insight into the field, it may also lead to certain expectations and preconceived understandings. By virtue of these preconceptions and our experiences as researchers and clinical nurse specialists, we are thus part of the interpretation: this is what Gadamer [14] refers to as the hermeneutic circle. We continuously challenged these assumptions throughout the research process through ongoing reflection and dialogue with one another. In this study, we aimed to gain a deeper understanding of patient experiences of the reasons for their prolonged hospitalisation. Our research process, analysis and interpretation of the transcribed interviews were guided by Gadamer’s philosophical hermeneutics approach. The study adhered to the Consolidated Criteria for Reporting Qualitative Research guidelines (Supporting file 1) [15].

### 3.2. Ethical Considerations

Carried out in accordance with the Declaration of Helsinki II, the participants received written and oral information about the objective of the study. All participants were informed of the option of withdrawing at any time with no implications for future treatment. Before the interviews began, participants were guaranteed anonymity and provided written consent. The transcribed interview files and sensitive patient data were stored according to applicable guidelines. The Danish Data Protection Agency approved the study (no. P‐2020‐295).

### 3.3. Study Setting and Recruitment

The study was conducted at the Department of Thoracic Surgery, Copenhagen University Hospital, Rigshospitalet, which has the largest thoracic surgery unit in Denmark, with 36 beds for patients with elective and acute conditions affecting the lungs, airways and oesophagus. Most patients are admitted for elective surgery for lung cancer and undergo VATS lobectomy, with the surgical trajectory structured around ERAS guidelines. This means that, in our local setting, patients attend preoperative consultations with a nurse, a surgeon and an anaesthesiologist, during which the expected treatment pathway is outlined. Patients are informed that the expected length of hospital stay after surgery is around 2 days, with the understanding that medical complications may occur and may potentially prolong the hospital stay. During surgery, a chest tube is inserted, and postoperatively, patients are to receive adequate analgesia, be mobilised and be provided with sufficient nutrition. When the chest tube no longer produces fluid or air, it is removed. Two hours later, a chest X‐ray is performed. If the findings are satisfactory, the patient can be discharged and scheduled for outpatient follow‐up and histological results 2–3 weeks later.

The first author recruited the participants included in the study. At the hospital, the individual participants were told about their rights, and the process of informed consent was explained. The participants were invited to take part in an interview, and those who consented were asked to sign a consent form. An interview was subsequently scheduled or held right away, whichever was more convenient. The first author, an experienced researcher who was not involved in the participants’ care or treatment, conducted the interviews. The aim was to include participants who had undergone VATS lobectomy and who had been hospitalised for longer than the medically recommended period. Other inclusion criteria were that the participants had to be able to speak and read Danish and had to be at least 18 years old. Exclusion criteria were dementia or any other mental health condition/issue affecting the brain. Between May 2020 and December 2021, 11 patients were invited to participate, of whom 9 agreed and 2 declined (Table 1). The interviews were held in a quiet room at the hospital while the participants were admitted. The audio‐recorded interviews were later transcribed verbatim, and the interviews lasted from 18:10 to 53:09 min.

**TABLE 1:** Participant characteristics.

ID^a^	Sex	Participated	Age (years)	Days in hospital	Family member	Length of interview (minutes)
Participant 1	Male	Yes	76	3	Lives with partner requiring care, contact with children	25
Participant 2	Male	Yes	80	4	Lives with partner, contact with children	25
Participant 3	Female	Yes	74	3	Lives alone, contact with children	18
Participant 4	Male	Yes	74	7	Lives alone, contact with children	19
Participant 6	Male	Yes	72	3	Lives with partner requiring care, contact with grandchild	25
Participant 7	Male	Yes	64	8	Lives alone, contact with children	23
Participant 8	Female	Yes	76	3	Lives with partner, contact with son	28
Participant 9	Male	Yes	64	3	Lives alone, contact with daughter	53
Participant 10	Female	Yes	86	3	Lives alone, contact with daughter and grandchildren	35

^a^Participants 5 and 11 said no to participate.

### 3.4. Data Collection

In Gadamer’s philosophical hermeneutics, humans are understood as linguistic beings: Language is our means of comprehension; therefore, dialogue is an important part of understanding [14, 16]. To gain insight into the patients’ experiences of their extended hospital stay and proceeding from the theoretical standpoint of the study, unstructured interviews were chosen as the data collection method. The unstructured interview is the method that best emulates an actual conversation since researchers are not constrained by predetermined questions and have the scope for a more uninhibited and spontaneous exchange about participant experiences [17]. Gadamer writes that a true conversation is driven by a basic desire to get closer to the other person. This does not occur through understanding the person themselves but through understanding what they say, i.e., understanding the matter [14]. Hermeneutic understanding is therefore a dialogical understanding, in which it becomes possible through conversation to gain a new understanding of the matter and thus reach a fusion of horizons [14, 16]. The unstructured interviews were designed to obtain detailed descriptions of participant experiences and investigate the areas which the participants felt were important and which they highlighted and emphasised in their understanding of why they had stayed in hospital longer. For this reason, the interviews were not structured around a preestablished interview guide. Prior to the interviews, the authors reflected together on how to formulate the questions as openly as possible and during the interviews, we remained aware that our clinical background, including our preunderstandings, could influence the questions we asked. The interviewer furthermore ensured that the interview remained in line with the objective of the study by asking open and exploratory questions about areas participants had identified. The interviewer started the interviews with variations on the question, ‘What do you think the reasons are for you staying in hospital?’ The point of this question was to get the participants to reflect on their situation and then talk about how they felt about their situation. Elaborating and clarifying questions were asked during the course of the interviews to flesh out participant responses. The sample size was determined based on Malterud’s concept of information power, which suggests that the more relevant information the sample holds for the study aim, the fewer participants are needed [18]. We initially aimed to include 10 patients to achieve sufficient information power; however, this was not possible due to limited eligibility and two patients declining to participate. Consequently, nine patients were included in the study. After transcription and preliminary analysis, the data were considered sufficient due to the specificity of the study aim and the in‐depth quality of the interviews.

### 3.5. Data Analyses and Interpretation

Within Gadamer’s philosophical hermeneutics (2007), understanding is ontologically tied up with preunderstanding, prejudice, language, tradition, history and application, which are closely linked to Aristotle’s concept of phronesis (practical wisdom, e.g., the ability to figure out what is good understanding, rather than true understanding) [19]. According to Gadamer, this link is inescapable; thus, we as researchers are always part of the research field being investigated, which means that our knowledge and preunderstandings are tethered to the way in which it is understood and interpreted. The objective of this study was to explore the experiences of causes of prolonged hospitalisation in treatment trajectories where no medical reason was identified, and the analytical process was structured as follows: First, the two authors read through the transcribed interviews to gain a general overview of the content of the texts. We then read the texts multiple times to identify recurring themes in the nine interviews, indicating the experience of causes of prolonged hospitalisation. We discussed the data and wrote down meaningful quotations for each theme. Finally, we reread the quotations and gave each individual theme a heading¸ continuously moving between the parts and the whole to refine our understanding. This cognitive process was circular and took place in the relationship between the subject and the researchers as interpreters, reflecting the hermeneutic circle, which in principle is an infinite and unending process [14]. Philosophical hermeneutics does not offer any recipe for how to create a correct interpretation process, but Gadamer [14] does describe how we, as researchers, in a process of interpretation, must guard ourselves against arbitrary whims and the limitations of unheeded habitual thinking and instead direct our attention to the matter itself. We sought to do this by staying focused on the matter and engaging in reflective dialogue, continuously revising our own version of understanding. Through this process, meaning was deepened while preconceptions and preunderstanding extraneous to the matter perishes [14]. This continuous reflective process resulted in three themes: *Role as patient or caregiver, Ready to be discharged* and *Relatives as a shield against worry*, which are presented in detail in the next section.

## 4. Findings

This section, which presents the findings of the study, is divided into three subsections: Role as patient or caregiver, Ready to be discharged and Relatives as a shield against worry. The subsections analyse the experiences of the participants in relation to the objective of the study, namely, to investigate why the participants have stayed in hospital longer despite having completed their medical treatment programme.

### 4.1. Role as Patient or Caregiver

This section shows the various ways in which the participants experienced their situation both as a hospitalised patient and as someone with responsibility for family members at home. It casts light on how being responsible for a partner who is ill can create a dual burden for the participants, who must manage their own illness while simultaneously being the primary support person at home. The study participants who live with a sick partner describe how they must manage some of the caregiving for their partner daily in addition to household chores, such as shopping and cleaning. Participant 1, whose wife had a brain haemorrhage and is wheelchair‐bound, describes how he thinks it is completely natural for him to take care of everything but that, at the same time, it is generally difficult because of his bad back and that putting a positive spin on it is hard. While Participant 1 is in hospital, his adult children have taken over the chores at home and the care of his wife. He explains how his children think he should deal with his stay in hospital:

*“My children said: ‘Just pretend it’s a holiday’. I wouldn’t say it’s a holiday exactly, but I am taking it easy*.*”* (Participant 1)

This quote can be seen as an attempt to minimise or divert attention from the real and possibly overwhelming challenges he faces with a recent lung cancer diagnosis and as a caregiver to his sick wife. His children have tried to ease his feelings by encouraging him to have a break and relax, but the participant is doubtful that his hospital stay could really be described as a break. This ambivalence may indicate an inner conflict between his need to focus on himself, his recent diagnosis and his need to recover from lung cancer surgery, on the one hand, and his feeling of obligation toward his partner and his family, on the other. The practical and emotional burden of his caregiver role at home makes it difficult for him to let go of his responsibility and concentrate only on himself. This could be a reflection of a feeling of inadequacy with regard to his role as a family member and as a patient. Thus, we can see how remaining in hospital, despite everything, may feel like a break but not necessarily a true respite from responsibility.

Participant 6 also has a partner at home who is seriously ill and has been for a number of years. He likewise describes how he is the one who sees to the household chores and the caregiving. His wife’s care is palliative, and he describes how he feels close to exhaustion and is ‘just about hanging in there’. His adult grandchildren are looking after his wife while he is in hospital. After surgery, he feels less able than before to cope with his duties at home, and when asked during the interview whether he knows why he is still in hospital, he replies:

*“As I understood it, they assumed I would be going home today, which is really soon. But I just couldn’t cope … So I’m still here. Because I asked to stay … I need to get my strength back… I can’t go back home to my wife and be a burden*.*”* (Participant 6)

This participant clearly describes his concern about being a burden to his wife if he is discharged too soon. His wish to stay in hospital ‘to get my strength back’ may be an indication that he does not feel up to managing both his own recovery and the care of his wife. This feeling could be linked to a fear of not being able to fulfil his obligations and could be an important reason for his wanting to stay in hospital. When the participant sees himself as having a dual role as patient and caregiver, the role of a strong, responsible husband who takes care of his wife is challenged when his own health is not good enough to be able to cope with these tasks. This creates a conflict between the need for his own recovery and the desire to maintain the role of the person with responsibility in the home. Such a conflict can lead to feelings of guilt and may possibly also undermine the participant’s self‐image. This challenge of wanting to maintain a role, with the ability to make a recovery being viewed as a prerequisite for fulfilling the caregiver role for a sick partner, underlines the difficult balance between the patient’s own health needs and family responsibility. Not being strong enough to care for a sick partner can thus be linked to a sense of loss of control over the role of a resourceful family member.

These examples of the dual role of being at once patient and caregiver show how being a patient in an ERAS programme for lung cancer extends well beyond surgical intervention and healing of the physical body. The participants’ illness and treatment are seen from their perspective as part of their overall life situation, which is characterised by the multiple roles that make up their life and, as shown in the case of these participants, by a responsibility that means that others are dependent on their help and support. Thus, they need the physical and mental energy to be there for someone and fulfil their caregiver role, while they themselves are ill, vulnerable and dependent on help and support—something that can be hard to get to grips with on the first planned day of discharge from hospital. Prolonged hospitalisation can be beneficial as it can provide respite and relief from the burden of responsibility—the opportunity to simply be oneself without having to think about others’ needs and without the responsibility that comes with that. Thus, an additional day at the hospital allows the patient to recuperate in a responsibility‐free framework. The two quotations above demonstrate that, when the participants’ overall life situation is taken into consideration, there may sometimes be a need to prolong hospitalisation. The responsibility that the participants bear at home rules out the possibility of recreation time in their home environment as circumstances dictate that they must function as a supporting caregiver to their partner.

### 4.2. Ready to Be Discharged

The following illustrates the problems that can occur when hospital discharge procedures are standardised and also highlights the social and communication aspects of discharge. The analysis shows how the process of being discharged from hospital, whereby healthcare professionals primarily follow standard procedures rather than considering individual needs and wishes, can seem from the participants’ perspective to involve a certain automatism.

The standardised hospitalisation procedure has a fixed structure. Depending on the X‐rays, patients may go home on the same day as the drainage is removed, and some participants state that they find this very early following lung surgery. Participant 9, who had lung cancer surgery and lives alone, describes how he felt when he learnt that he was soon to be discharged:

*“They told me yesterday: ‘You need to go down to have an X-ray before you go home.’ But me being able to go home or not doesn’t depend on that X-ray. It’s like you have to have an X-ray so we have proof that you can go back home.”* (Participant 9)

In the standardised ERAS procedure, the expectation is that patients can return home as soon as they have their drainage removed. They have an X‐ray 2 hours after the drainage is removed, but instead of waiting for the results of the X‐ray, the expectation is conveyed that the patient can go home even before the X‐ray is taken and examined. From the patient’s perspective, it can feel as though the X‐ray is taken more to confirm the decision to discharge them from hospital rather than to actually examine how they are. As the example above shows, the participant reflects on how having the X‐ray feels more like a formality to merely ‘prove’ early discharge rather than to genuinely assess his condition. This is an illustration of the automatism that occurs in the discharge procedure, where the individual needs of the participant and their condition seem to be overlooked when the standard procedure is followed. Thus, it becomes a process that simply has to be gone through rather than an assessment of whether the participant is actually ready to be discharged. In this sense, being discharged from hospital can come across as something done for the sake of a standard procedure rather than the individual’s best interests. Patients can thus feel reduced to a cog in the wheels of a standard system that disregards individual needs and understanding. This kind of approach amplifies a sense of lack of autonomy and control, challenges communication and fosters uncertainty about the decision to discharge the patient.

Another participant describes his experience of being told that he would be discharged:

*“… she just came in [the nurse] and said: ‘Right [name of participant 2], everything is okay so you can go home now’. It was like that. They didn’t ask me if I had any symptoms or anything like that*.*”* (Participant 2)

This situation illustrates how the participant’s experience of their body, symptoms and health can feel subordinated to a standardised procedure. The quotation indicates the participant’s expectation that the healthcare staff should be interested in his symptoms before he is discharged. However, he feels that this is not the case and that communication about the decision to discharge him is based on a standard set of measures according to which he may go home if the X‐ray is good—an approach which makes him feel unseen and not involved in the decision. This is an instance that demonstrates how standardised procedures and automated decision‐making can lead to patients feeling passive in the process.

To what extent healthcare professionals are attentive to the individual hospitalisation needs of the patients varies. Some participants, for example, have to specifically request to stay in hospital longer. One participant stated:

*“… it was a bit blunt, the way he said it: ‘Right, you can go home today’*. *But you know what, I live on Bornholm [an island far away from the hospital]*.*”* (Participant 8)

For this participant, the question of extending her stay in hospital is embarrassing, and she describes how she was reluctant to ask if she could stay longer. She describes feeling pressured to leave without having the chance to make arrangements for making the long journey home to Bornholm safely and comfortably. An experience like this can lead a patient to feel insecure and emotionally pressurised; the participant faces practical challenges which, from her perspective, the nurses are not taking into proper consideration. She may also feel overlooked because her needs have not been understood or acknowledged. Once the participant articulated her concerns to the healthcare professionals about being discharged, however, she remained in hospital until her husband could accompany her and she felt confident about going home. This example demonstrates how easy it is for healthcare professionals to lose sight of a patient’s overall situation, also illustrating the automatic nature of the decision‐making procedure for hospital discharge after the drainage has been removed. Expecting patients to explain the situation they are in and to take responsibility for their needs by communicating what they require to feel safe when discharged from hospital is asking a great deal. It requires patients to actively engage in the decision to discharge and means that they must have the opportunity to express their health‐related needs and concerns.

Despite the standard procedure of discharging patients on the day that their drainage is removed and despite the fact that some of the participants want to go home, some of them remain in hospital. During an interview, one participant explains:

Participant 7: *“I just don’t see why I need to be here. I’d rather go home …”*

Interviewer: *“Who do you think made that decision?”*

Participant 7: *“Presumably the senior consultant.”*

The participant explains later in the interview that he was told that his pain treatment had to be adjusted, but he also explains that he is somewhat puzzled because he does not feel any pain. Thus, there is a lack of clarity about the precise reasons for deciding that the patient should remain in hospital. It seems that the healthcare professionals have decided to extend his stay in hospital, yet the patient does not feel involved in the decision, does not understand why he is in hospital and indicates that he is ready to go home. Another participant describes her experience of prolonged hospitalisation:

Participant 4: *“Yesterday he [the doctor] said that it [discharge] might be on Tuesday or maybe Wednesday*.*”*

Interviewer: *“Do you know why? What the reasons are? Did he say what they were waiting for?”*

Participant 4: *“No, but I didn’t mind. Wednesday sounded fine to me.”*

It is decided, for unknown reasons, that this participant should remain in hospital, and the patient is not informed about or cannot recall the reason why he is supposed to stay.

The above quotes and analysis show how healthcare professionals in some instances almost automatically suggest discharge from hospital on the day of drainage removal. Thus, the onus is on the patients to ask to remain in hospital if they feel nervous about going home. At the same time, there are other situations where healthcare professionals decide to prolong hospitalisation without the reasons being clear to the patient. The quotations above show that this may be accepted but also may be a cause for confusion.

### 4.3. Relatives as a Shield Against Worry

This section presents different ways in which family members can provide relief and play a protective role in addressing the participants’ concerns, both before and after discharge, and shows the distinction between the practical and emotional support that family members provide. Many of the participants had concerns about how they would manage after being discharged, for example, what they had to do to take care of their health or their symptoms after surgery, or whether there was food in the fridge or how to get back home. One participant describes her worries before being discharged:

Interviewer: *“What do you feel unsure about?”*

Participant 10: *“About whether I’ll have pain or there’ll be any bleeding or whatever … if I suddenly take a turn for the worse during the night, I don’t have anyone to call on.”*

Her worries already start while she is in hospital and, as the quotation above shows, they can be worries relating to symptoms and how much the body is capable of after surgery. At the hospital, the participant notices that her body feels different, that she has pain and that she is not able to do what she could do earlier. Her worry both relates to what she is experiencing in the moment and to whether her body can manage day‐to‐day tasks once she has been discharged. During an interview, one participant is asked to explain in his own words why he is still in hospital:

*“Because yesterday I didn’t feel that I could go home to my first-floor flat. I didn’t have anyone to help me yesterday.”* (Participant 9)

What the participants are articulating can be understood to be concerns relating to the transition from hospital to home, where they lose the constant presence of healthcare staff and feel unsure about how their bodies will react after the surgery. There is an existential dimension here, in that the patients feel vulnerable with respect to being confronted by the unknown when they have to take responsibility for their own care. As both examples show, a significant factor in how confident the participants feel when it comes to being discharged is whether they live alone or have relatives who can offer help. In the first quotation, the worry is about not having anyone to call on if the participant suddenly takes a turn for the worse, while the second quotation is more about practical help to get up the stairs to the participant’s flat. Thus, we see how the participants have varying needs for support and how relatives can act as a shield against worry and as a buffer against the unknown. All the participants in the study have a social network (Table 1), and they describe in the interviews how their family members are willing to help in various ways. The interviews clearly show that family members are an important aspect of whether the participants feel safe returning to their home. One participant describes her view on being discharged and having a family member at home as follows:

*“If I had a husband waiting for me at home and I went back, that would be all right. Someone would be there for me. But when you’re all on your own, it can be a bit frightening, especially at night*.*”* (Participant 10)

Not only does the role of family members go beyond practical help, but, as the quotation also shows, having someone at hand can ward off worries and feelings of anxiety. In the interview, the participant, when asked, says she has not felt any anxiety while in hospital because someone regularly comes in to check on her. Therefore, we can interpret her experience as relating to a feeling of fundamental insecurity and concern about being alone and taking care of herself.

## 5. Discussion

In a national and international context, the focus is on optimisation, rapid assessment, diagnosis, treatment and a brief hospital stay, thus accelerating the patient process, which from an overall perspective is positive for treatment times and ultimately survival. However, in clinical practice, we sometimes encounter patients whose life situation clashes with standardised programmes and a scheduled quick discharge from hospital despite the fact that they have had surgery and ought to be ready to leave the hospital. Our aim with this study was to better understand these patient trajectories by examining the various possible dimensions of patient experiences of prolonged hospitalisation.

### 5.1. Role as Patient or Caregiver

In the first section of the analysis, which looks at the participants’ role as patient or caregiver, we found that the participants who fulfil both the role of patient and that of a family member acting as a caregiver for another family member [20] may need to stay in hospital longer than the typical standard period for a lung cancer surgery programme. The analysis furthermore indicates that the participants’ situation can be described as caregiver burden, which is defined as ‘*the strain or load borne by a person who cares for a chronically ill, disabled, or elderly family member*’ ([21], p. 439). In a scoping review examining roles and experiences of informal caregivers, it has been found that informal caregivers have a huge responsibility when it comes to navigating the health system. They play an important coordinating role in trying to ensure, as best they can, continuity in the treatment and care of their relatives in what is often a fragmented and highly specialised system [21]. Their support is therefore crucial in the processes involving the people for whom they are caregivers. Furthermore, research on the concept of caregiver burden has found that people who are caregivers for a sick family member often experience decreased care provision, decreased quality of life and deterioration of physical and psychological health [21]. The consequences of caregiver burden can thus be wide‐ranging for the individual. When the participants in this study explain that they cannot view their hospital stay as a break or cannot go home to their partner and be a burden to them, this is an indication that the role of caregiver can never be shed, not even if the individual has a cancer diagnosis or is recovering after surgery. Patients who are informal caregivers live with the duties and consequences that their role entails, even if they are afflicted by a serious illness. Even though standardised ERAS programmes require patient cooperation [22] to ensure that they are on board with the premises of the standardised programmes, including early discharge from hospital, patient circumstances may nonetheless dictate a different approach to hospitalisation to ensure that the patients go through surgery successfully; an additional day in hospital may give them the extra time to recover in a setting allowing them to be patients rather than caregivers. As healthcare professionals, we need to be aware of the patients’ general life situation and of the fact that some patients have circumstances at home that require us to diverge from standard times for hospital discharge. One way of structuring this is through person‐centred care, which focuses on recognising individuals’ capabilities and potential to manage and improve their own health [23]. This can be achieved through a focus on narrative communication, where the person’s experiences, perspectives of their life situation and health condition are consistently placed at the centre of care [24]. A study comparing person‐centred care with standardised care among patients undergoing total hip arthroplasty even found that the person‐centred approach was associated with a reduced length of hospital stay [25].

### 5.2. Ready to Be Discharged

One study exploring the perspective of thoracic surgery nurses with regard to extended hospitalisation after surgery showed that the nurses sometimes adjust the standard hospitalisation time in order for the patients to successfully get through the postsurgerical process [13]. The article described how parallels can be drawn between the ERAS programme and an organisational production paradigm in which nursing practice is compared to an assembly line and is primarily based on fixed guidelines. Norlyk et al. [26] drew similar parallels with a production paradigm, describing how standardised procedures involving one best way potentially lead to a McDonaldisation of nursing practice. The study by Missel et al. [13] also showed that nurses should actively engage with each individual patient to ensure that the patient is truly ready to be discharged—not just blindly follow standardised guidelines. A similar point is made by Sibbern et al. [27] regarding individualised care versus a standardised ERAS programme. Their findings show that patients identified healthcare professionals’ support and personalised behaviours as a decisive factor in their active engagement with the objectives of the ERAS programme. In this present study, we can see how the participants feel listened to by the healthcare staff when they express the need to stay longer in the hospital, although they have to independently initiate and articulate this need, which is not easy for many of them and can be a definite challenge for some. At the same time, this study has found that some participants are ignorant of the reasons why their hospitalisation has been extended. Possibly, in such cases, the healthcare staff have made a professional assessment and decided that a particular patient needs to stay in hospital for a longer period, but they have not actually informed the patient about the decision. Above all, clear and regular communication during hospitalisation is important for health professionals to gain the patients’ trust [28] and for the patients to develop a coping strategy during prolonged hospitalisation [5]. The same seems to apply to the findings in this study that show that some participants are confused about decisions to end or prolong hospitalisation and do not always know the reason for deciding one way or the other. Furthermore, this study shows that the participants desire a shift in focus so that X‐rays are not used as documentation to determine that a patient may go home but rather as a basis for ascertaining how the patient is doing. In addition, the participants would like greater focus to be placed on their overall life situation, with their experience of symptoms and the transport distance from hospital to home being considered in the decision about when to discharge them. This can be taken as an indication that the participants would like the consultation about early discharge to be more individualised. Another study has shown how a patient‐centred discharge consultation in which the nurse assesses the patient’s and the family’s individual needs can indeed improve patient and caregiver satisfaction and prevent readmissions [29]. In their study, Rojas‐García et al. [10] found that prolonged hospitalisation without specific medical causes can have several negative consequences for patient well‐being. Our study enhances this perspective by adding that extended hospitalisation in cases where there is no medical cause can be important for a patient’s well‐being and may allow them to feel secure and thus ready to be discharged.

### 5.3. Relatives as a Shield Against Worry

The third section of the analysis shows how the participants can experience all kinds of worries in connection with their imminent discharge from hospital after lung cancer surgery. The participants who live alone can experience a different type of concern related to the fact that no one is at hand to provide support and they are dependent on their relatives to help with things like grocery shopping. Earlier research has shown that readiness for discharge is connected to feeling safe; patients who felt safe considered themselves ready to be discharged [12]. Furthermore, studies have shown that family members and friends play an important role after patients have been discharged [27, 30]. In this study, we find that family members are already highly important before discharge when the participants envisage returning home; they are important both on a practical level and in terms of providing psychological support by just being there. Being alone, or simply the thought of being alone, is very worrying as the time for discharge approaches. The findings of this study show that relatives play a significant part in patients’ recovery within ERAS programmes. These findings are consistent with those reported by Sibbern et al. [27] in their systematic review of qualitative studies. This means that there is a risk that those patients who do not have friends or family members to provide support are at a disadvantage. It is imperative that healthcare professionals are aware of their situation and can navigate the standardised procedures so that these patients also feel safe when discharged from hospital. This might mean, for example, longer hospitalisation or a telephone call by the nurse after discharge [30]. The healthcare professionals must view the patients from their particular life situation and plan what they need from there. Patients live in different circumstances, and an individual, professional assessment and, if relevant, differentiation in hospitalisation periods may be necessary to ensure that all patients successfully go through their treatment programme and recovery.

### 5.4. Strength and Limitations

To the best of our knowledge, the question of why patients remain in hospital despite having recovered physically after lung cancer surgery has not previously been examined from the patients’ perspective. Although ERAS programmes have been implemented worldwide in a number of specialities [2], we still lack knowledge about possible unintended consequences arising from rigidly structured nursing guidelines. This article is a contribution to the steadily growing body of knowledge within the field.

It was not easy to recruit patients for this study. Very few patients stay in hospital longer despite having recovered physically. Huang et al. [4], for example, recruited around three patients during the inclusion period in their study, corresponding to 2% of the 147 patients asked. This dynamic is also reflected in the present study. It was difficult to recruit patients since not many met the inclusion criteria. Over a roughly 19‐month period, we managed to identify 11 patients who could potentially be included, of whom 9 agreed to take part. We would like to stress, however, that notwithstanding the small number of included patients, the knowledge gained about this patient group is valuable.

During the interview process, we found it difficult to ask the participants why they were still in hospital. We adopted a cautious but open approach but sensed a certain incomprehension vis‐à‐vis the question. The participants generally indicated that they considered the hospitalisation very brief. This discrepancy between how the participants generally perceived the length of hospital stay as short and our understanding of it as prolonged was difficult to address in the interviews. These varying perceptions are a relevant area for further investigation in future studies.

As clinical nurse specialists and researchers, we represent an institution, which likely influences the dynamics during the interview. In Section 3 of the methodology, we sought to provide insight into the current context in which we work and the preunderstandings that inevitably accompany us. We have outlined the aspects that were accessible to our awareness and reflection while recognising that it is impossible to fully account for all dimensions of our preunderstandings. The extent and manner in which this impacted the study’s results remain unclear. Gadamer [14] explains that the researcher is seen as an active participant in the interpretation process, which implies that others might have interpreted the interview statements differently. Throughout our analysis, we have aimed to be transparent by using quotes from the interviews. This enables the reader to assess the credibility of what is presented and whether other analyses could be more plausible [32].

### 5.5. Implications for Practice

The findings can be used in clinical practice to improve our understanding of why some patients undergoing surgery for lung cancer in an ERAS programme need to remain hospitalised despite having completed the treatment medically. Nurses should pay attention to identifying patients experiencing caregiver burden early in the care trajectory. This could be addressed during the preoperative consultation with a narrative approach inspired by person‐centred care. Early recognition of the individual patient’s life circumstances may support the development of a more individualised care trajectory. The study findings underline the importance of nursing that is not exclusively based on standardised guidelines when planning hospital discharge but which takes as its starting point the patients’ individual needs and overall life situation. Future studies should continue to investigate the extent to which standardised programmes influence nursing care.

## 6. Conclusion

This study has shown that patients who have undergone lung cancer surgery in an ERAS programme can often be discharged as planned. However, some patients are in such different situations that it is not possible for them to meet criteria for a standard time for hospital discharge. From the study findings, we can conclude that patients who are providing care for a partner at home occupy a dual role as patient and caregiver alike, which means that they are caught between the need to recover from a serious diagnosis and surgery and responsibility for taking care of a sick partner. In this case, the time of discharge can have relevance when it comes to the patients’ home circumstances. We can furthermore conclude that it is easy for healthcare professionals to lose sight of the patients’ general situation and the fact that standardised procedures and automated decision‐making can take over and make the patients feel that they are not involved in the decision to discharge them from hospital. Thus, hospitalisation can end up being shorter or longer than they wish. Furthermore, the study has shown that relatives can create a sense of security and be a shield against worry both before and after discharge. Thus, the time of discharge could depend on the availability of a family member to provide support in the form of hands‐on practical help or being reassuringly nearby to offer support.

All in all, this study shows that there is a small group of patients in the ERAS programmes who need the pace of the procedures to be slowed down so that they can feel ready to be discharged. Healthcare staff need to be aware of the patients’ general life situation and need to be able to diverge from the standard when necessary. Thus, the time of discharge should be seen in a larger context that takes into account the individual patients’ unique life situations.

## Funding

This research received no specific grant from any funding agency in the public, commercial or not‐for‐profit sectors.

## Conflicts of Interest

The authors declare no conflicts of interest.

## Supporting Information

Additional supporting information can be found online in the Supporting Information section.

## Supporting information


**Supporting Information** The study adhered to the Consolidated Criteria for Reporting Qualitative Research (COREQ): a 32‐item checklist for interviews and focus groups [15]. Please see Supporting file 1 for a detailed description.

## Data Availability

The study data are available from the corresponding author upon reasonable request that does not conflict with keeping the data anonymous and confidential.
